# Revisiting distinct nerve excitability patterns in patients with amyotrophic lateral sclerosis

**DOI:** 10.1093/brain/awae131

**Published:** 2024-04-25

**Authors:** Diederik J L Stikvoort García, H Stephan Goedee, Ruben P A van Eijk, Leonard J van Schelven, Leonard H van den Berg, Boudewijn T H M Sleutjes

**Affiliations:** Department of Neurology, Brain Centre Utrecht, University Medical Centre Utrecht, Utrecht, 3584CX, The Netherlands; Department of Neurology, Brain Centre Utrecht, University Medical Centre Utrecht, Utrecht, 3584CX, The Netherlands; Department of Neurology, Brain Centre Utrecht, University Medical Centre Utrecht, Utrecht, 3584CX, The Netherlands; Biostatistics and Research Support, Julius Centre for Health Sciences and Primary Care, University Medical Centre Utrecht, Universiteitsweg 100, 3584CX, Utrecht, The Netherlands; Department of Medical Technology and Clinical Physics, University Medical Centre Utrecht, 3584CX, Utrecht, The Netherlands; Department of Neurology, Brain Centre Utrecht, University Medical Centre Utrecht, Utrecht, 3584CX, The Netherlands; Department of Neurology, Brain Centre Utrecht, University Medical Centre Utrecht, Utrecht, 3584CX, The Netherlands

**Keywords:** ion channels, amyotrophic lateral sclerosis, progression rate, composite measures, nerve excitability, EMG

## Abstract

Amyotrophic lateral sclerosis is a devastating neurodegenerative disease, characterized by loss of central and peripheral motor neurons. Although the disease is clinically and genetically heterogeneous, axonal hyperexcitability is a commonly observed feature that has been suggested to reflect an early pathophysiological step linked to the neurodegenerative cascade. Therefore, it is important to clarify the mechanisms causing axonal hyperexcitability and how these relate to the clinical characteristics of patients.

Measures derived directly from a nerve excitability recording are frequently used as study end points, although their biophysical basis is difficult to deduce. Mathematical models can aid in the interpretation but are reliable only when applied to group-averaged recordings. Consequently, model estimates of membrane properties cannot be compared with clinical characteristics or treatment effects in individual patients, posing a considerable limitation in heterogeneous diseases, such as amyotrophic lateral sclerosis. To address these challenges, we revisited nerve excitability using a new pattern analysis-based approach (principal component analysis). We evaluated disease-specific patterns of excitability changes and established their biophysical origins. Based on the observed patterns, we developed new compound measures of excitability that facilitate the implementation of this approach in clinical settings.

We found that excitability changes in amyotrophic lateral sclerosis patients (*n* = 161, median disease duration = 11 months) were characterized by four unique patterns compared with controls (*n* = 50, age and sex matched). These four patterns were best explained by changes in resting membrane potential (modulated by Na^+^/K^+^ currents), slow potassium and sodium currents (modulated by their gating kinetics) and refractory properties of the nerve. Consequently, we were able to show that altered gating of slow potassium channels was associated with, and predictive of, the rate of progression of the disease on the amyotrophic lateral sclerosis functional rating scale. Based on these findings, we designed four composite measures that capture these properties to facilitate implementation outside this study.

Our findings demonstrate that changes in nerve excitability in patients with amyotrophic lateral sclerosis are dominated by four distinct patterns, each with a distinct biophysical origin. Based on this new approach, we provide evidence that altered slow potassium-channel function might play a role in the rate of disease progression. The magnitudes of these patterns, quantified using a similar approach or our new composite measures, have potential as efficient measures to study membrane properties directly in amyotrophic lateral sclerosis patients, and thus aid prognostic stratification and trial design.

## Teaser

Stikvoort *et al.* report that nerve excitability in amyotrophic lateral sclerosis is characterized by four unique patterns, each originating from changes in different membrane properties. Excitability changes related to altered gating kinetics of slow potassium channels are associated with, and predictive of, the rate of disease progression.

## Introduction

Amyotrophic lateral sclerosis (ALS) is a devastating disease characterized by progressive loss of central and peripheral motor neurons.^1^ Despite the clinical and genetic heterogeneity of the disease,^1^ hyperexcitability is present in central and peripheral motor neurons.^2,3^ It has been suggested that these changes in excitability represent a key pathophysiological step in the cascade leading to motor neuron death.^4,5^ Measures of peripheral nerve excitability are valuable because they facilitate more detailed electrophysiological evaluation and enable verification of target engagement of potential candidate compounds that can modulate membrane excitability.^6-10^ Therefore, it is imperative to determine which membrane properties drive these changes, whether these are unique to patients with similar clinical profiles, and to clarify their association with the neurodegenerative process.

Advanced neurophysiological techniques, such as nerve excitability testing, allow non-invasive assessment of nerve excitability properties by tracking threshold changes in a group of axons at one nerve site.^11^ These threshold changes are dependent on the underlying membrane properties, such as the resting membrane potential, voltage-gated ion channels and pumps. Interpreting the complex set of threshold changes and identifying their specific membrane properties from nerve excitability measures directly, however, remains challenging.^12^ Mathematical models of myelinated axons can help in the interpretation of nerve excitability recordings.^13-15^ These models are an excellent tool for testing hypotheses, for example, by predicting the effect of up- or downward expression of voltage-gated ion channels through simulations.^16,17^ However, back-estimation from measurements is challenging, because multiple solutions can yield similar excitability patterns, which can be exacerbated by noise in individual measurements. Consequently, membrane properties are often estimated using group-averaged excitability measures, which cannot be associated with clinical metrics or individual treatment responses. Considering ALS heterogeneity, new approaches are needed to gain a better understanding of the role of excitability changes in the pathophysiology of the disease and to leverage this valuable technique optimally for application in clinical settings.

In this study, we revisited excitability changes in patients with ALS using a new approach. Principal component analysis (PCA) was applied to the full set of nerve excitability measures from patients with ALS. Our aims were as follows: (i) to identify ALS-specific excitability patterns and their biophysical origins; (ii) to study the relationship between the newly identified excitability patterns and the clinical profiles of patients; and (iii) to develop new, simple, compound measures that reflect these identified excitability patterns and that could facilitate practical implementation.

## Materials and methods

### Ethical approval

The study protocol and patient recruitment procedure were approved by the medical ethics committee of the University Medical Centre Utrecht and performed in accordance with the Declaration of Helsinki. All participants provided written informed consent prior to participation.

### Participants

#### Patient recruitment

Patients with a suspected motor neuron disease were recruited prospectively during their first diagnostic work-up at the outpatient clinic of the University Medical Centre Utrecht, between August 2020 and January 2023. Patients with cognitive dysfunction that could hamper compliance with the study, coincidental active neuropathies or use of nerve excitability-altering medication (e.g. riluzole)^9,10,12^ were not eligible for participation. Diagnoses were established using the Gold Coast criteria after study participation and were validated ≥6 months after participation. Reference measurements were obtained from 50 controls without a history of median nerve disorders, who were recruited from a prospective population-based register in the Netherlands^18^ or from previous studies.^19,20^

#### Clinical and demographic characteristics of patients

To document the clinical patterns of patients, we obtained various measurements of disease stage, such as (sub)scores from the revised ALS Functional Rating Scale^21^ (ALSFRS-R), and measurements detailing the progression rate of disease, such as ΔFRS [(48 − ALSFRS-R at study visit) / duration from symptom onset to study visit in months] and prognostic risk profile from the European Network to Cure ALS (ENCALS) survival model.^22^ This prognostic risk score is based on the following eight variables: bulbar onset (yes/no), definite versus probable or possible ALS, diagnostic delay (in months), force vital capacity (as a percentage), ΔFRS, presence/absence of frontal temporal dementia and C9orf72 repeat expansion. These clinical data were all obtained on the same day as study participation. Survival was defined by the time in months between study participation or symptom onset until >23h of non-invasive ventilation, tracheostomy or death.

### Experimental protocol

#### Preparation and set-up

Compound muscle action potentials (CMAPs) were recorded from the abductor pollicis brevis after stimulation of the median nerve at the wrist, as described previously.^23^ The software QTRAC (QTRAC, Institute of Neurology, Queen Square, London, UK) was used for recording and stimulus control. Before the recordings, the examined arm was warmed using a water blanket (Norm-O-Temp and Maxi-Therm Lite; Cincinnati Sub-Zero LLC) for 30 min using a previously described warming procedure.^24^ The water blanket was kept in place to maintain the arm at 37°C, thereby minimizing temperature-induced variability during the recordings.

#### Nerve excitability recordings

We performed multiple excitability tests from the well-established TRONDNF-protocol^25^ in the QTRAC software, including: (i) the strength–duration test (relationship between stimulus charge and stimulus durations of 1.0, 0.8, 0.6, 0.4 and 0.2 ms); (ii) threshold electrotonus (threshold changes to ±20% and ±40% depolarizing and hyperpolarizing conditioning currents of increasing duration up to 100 ms); (iii) current–voltage relationship (threshold changes to long-lasting conditioning currents of 200 ms from +50% depolarizing to −100% hyperpolarizing); and (iv) recovery cycle (threshold changes after supramaximal stimuli at decreasing interstimulus intervals from 200 to 2 ms). These tests yielded 142 measures in total [threshold electrotonus, *n* = 106; current–voltage, *n* = 16; recovery cycle, *n* = 18; strength–duration time constant (SDTC) and rheobase]. For secondary analyses, we included a number of standard excitability measures, including: TEd_peak_, TEd_40–60ms_ and TEd_90–100ms_ (percentage threshold change to +40% depolarizing currents at peak, 40–60 ms and 90–100 ms), TEh_90–100ms_ (percentage threshold change to −40% hyperpolarizing currents at 90–100 ms), Fanning (TEd_90–100ms_ − TEh_90–100ms_), S2 (TEd_peak_ − TEd_90–100ms_), TEd_Undershoot_ and TEh_Overshoot_ (under- and overshoot of threshold changes after 40% depolarizing and hyperpolarizing currents), resting current–voltage (*I*/*V*) slope (slope between thresholds after −10% and +10% conditioning stimuli of 200 ms), refractoriness (percentage threshold increase at 2-ms interstimulus intervals), relative refractory period (RRP; in milliseconds), superexcitability and subexcitability (percentage peak threshold reduction and increase after supramaximal stimulation, respectively).

#### CMAP scan-derived motor unit number estimates

We recorded detailed stimulus–response curves (CMAP scans), in which the number of recruited motor units decreases by gradually reducing the stimulus currents from supramaximal to subthreshold (2 Hz, pulse width 0.1 ms).^26^ Motor unit number estimates (MUNE) were derived from the CMAP scans using the MScanFit tool (v.2) in the QTRAC software.^27,28^ These MUNE values were used to characterize the disease stage of the examined muscle.

### Analysis of excitability changes

**Figure 1:**
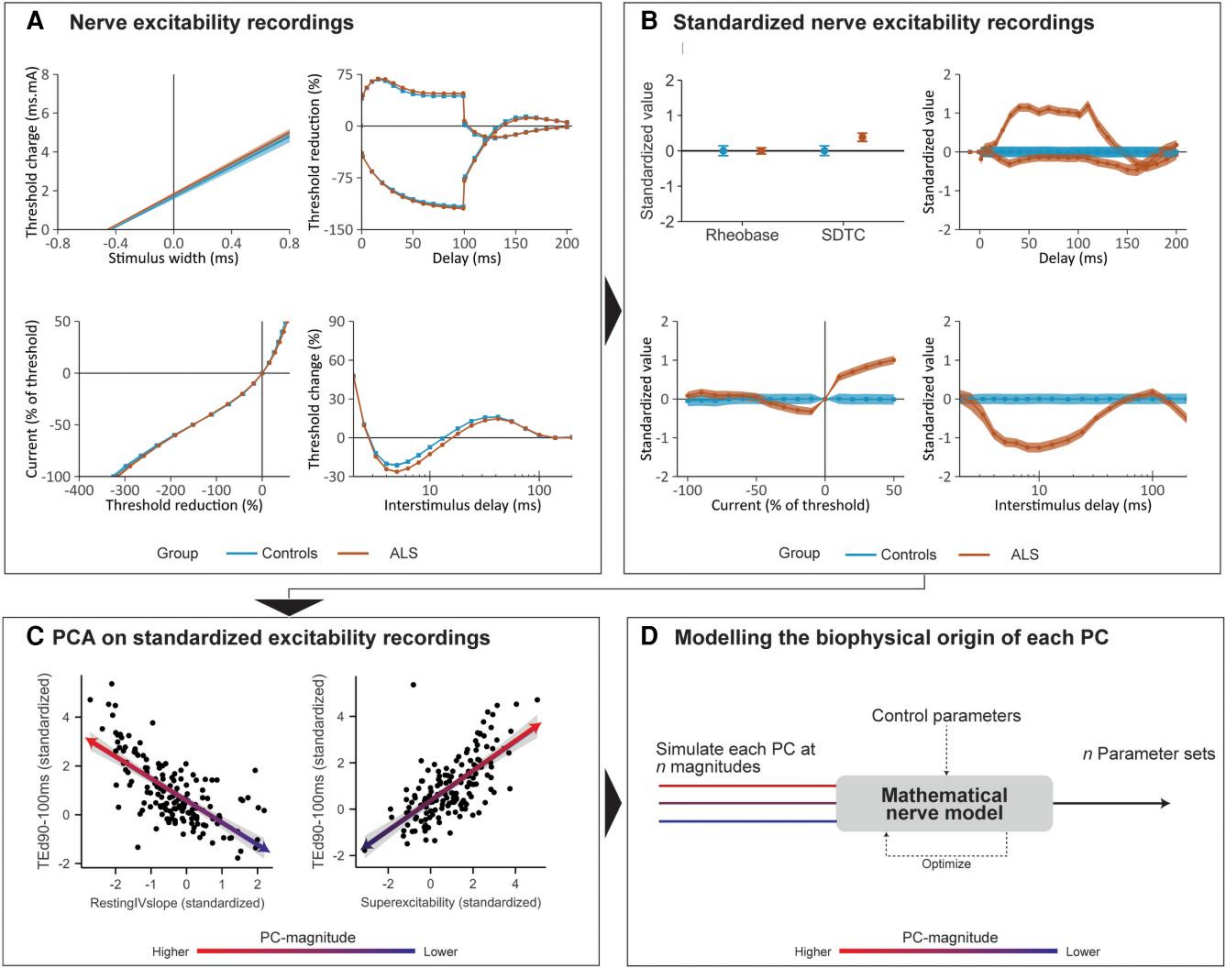
**Schematic overview of the analysis of excitability recordings.** (**A**) The four standard tests from the TRONDNF protocol in controls (blue) and ALS patients (orange), with, on the *top row*, the strength–duration test and threshold electrotonus and, on the *bottom row*, the current­­–voltage test and the recovery cycle. (**B**) Standardized and age- and sex-adjusted representation of the data in plot **A**, where each unit represents 1 standard deviation with respect to controls (blue; ALS patients are shone in orange); shaded areas and whiskers represent the standard errors. (**C**) Two correlation plots between standardized measures of excitability obtained from **B**, illustrating how the direction and magnitude of a potential PC (higher = red, lower = blue) with respect to the values of healthy controls capture patterns of changes in excitability. (**D**) A mathematical nerve model was fitted to excitability patterns that were simulated from each PC at *n* = 7 magnitudes, yielding *n* parameter sets for each PC to evaluate concordance of the optimizations. ALS = amyotrophic lateral sclerosis; PC = principal component; SDTC = strength–duration time constant.

The analysis of the excitability changes consisted of three main steps: (i) standardization of the measurements from ALS patients with respect to the control population to retain disease-specific effects; (ii) PCA on the resultant dataset to discern independent and distinctive excitability patterns; and (iii) application of a well-established mathematical model to clarify the biophysical origin of the observed excitability changes. An overview and illustration of these steps is presented in Fig. 1.

First, standardization was performed to ensure that the analyses were performed only on disease-specific excitability changes and not on those driven by age, sex, normal physiological variability or other potential confounders of excitability.^29,30^ Briefly, linear regression models were fitted to each excitability measure in the control population, with age and sex as potential covariates. Coefficients were bias corrected using 1000 bootstrap iterations to reduce the chance of overfitting. Using these coefficients, we obtained the residuals (e.g. observed minus predicted) for each patient, and we scaled these by the standard deviation of the non-neurological controls to account for the differences in magnitude at which the four nerve excitability tests operate (Fig. 1B).

Second, PCA was performed on the complete set of 142 standardized excitability measures from each subject. PCA reduces the dimensionality of large datasets by combining interrelated variables into a small subset of principal components (PCs), which are obtained for each individual in the population (Fig. 1C). Consequently, the resulting PCs reflected patterns of ALS-specific excitability changes, whereby larger deviations in the underlying measures yielded larger PC magnitudes. These PC magnitudes were the main outcome measures for comparing nerve excitability changes with clinical characteristics of patients. The optimal number of PCs to retain and their respective stability was evaluated using a cross-validation approach (Supplementary material).

Third, we explored which underlying biophysical properties were major determinants for the PC patterns of ALS-specific excitability changes. We used a two-compartment model (a node of Ranvier and internode) implemented in the software Qtrac-P, described in detail previously.^14^ This model incorporates several properties of key ion channels (conductance and gating kinetics), pumps and ionic concentrations that modulate axonal excitability. A detailed description of all model parameters is provided in Supplementary Table 1. First, we optimized the model to the mean excitability recordings of the control cohort (all PC magnitudes are zero), where the obtained parameter set served as reference when changing the individual PCs. The discrepancy between the measured and simulated excitability recordings was minimized using previously applied relative weights for each test: 0.5 (strength–duration), 2 (threshold electrotonus), 1 (current–voltage) and 1 (recovery cycle).^20,31,32^ The error was inversely weighted by the standard deviation at every measured threshold. The resting membrane potential was allowed to vary during optimization in response to parameter changes. Using these control parameters as the starting point, we then fitted the model onto each PC pattern that was simulated at various magnitudes (−15 to 15 in 5-unit steps; Fig. 1D). Parameters that consistently yielded accurate fits were considered to be a major determinant of the corresponding PC magnitudes. A median discrepancy reduction of ≥30% was deemed acceptable for the modulation of single parameters.^15,16^

### Composite excitability measures capturing the PCs

In clinical settings, such as trials or diagnostic visits, it might be beneficial to obtain a smaller set of measures derived from a shortened excitability recording, rather than a full recording, while maintaining most of the excitability variance between patients. Therefore, in order to describe the established ALS-specific patterns of excitability changes in an effective manner, we derived new composite measures using the standard excitability measures. We fitted a linear regression model for each PC magnitude, with all standard excitability measures as covariates. Then, a backward stepwise selection procedure was performed based on Akaike’s information criterion; this process was replicated 1000 times through a bootstrap procedure. Finally, we retained the five most frequently selected standard excitability measures and the corresponding coefficients.

### Statistical analysis

We used Mann–Whitney U-tests and Fisher’s exact tests to compare demographic data, clinical data and MUNE. Standard nerve excitability measures were compared between patients and non-neurological controls using a linear regression model, adjusting for age and sex.

Overall, the amount of missing data was minimal (1.2%), with the exception of measurements made during strong hyperpolarizing currents of the current–voltage test (22% missing at −100% conditioning). We accounted for missing excitability data using multiple imputation with chained equations (MICE; *n* = 20 imputed datasets). PCA was performed in each imputed dataset to minimize imputation bias. All subsequently described statistical tests were likewise performed in each imputed dataset and pooled using Rubin’s rules. Differences in the PC magnitude between patients and controls were assessed using Student’s *t*-tests. Pearson’s correlation coefficient (*r*) was used to evaluate the correlation between PC magnitudes and standard excitability measures. Linear regression models were used to quantify the relationship between PC magnitudes and continuous clinical characteristics.

To examine the association between PC magnitudes and survival in patients, we used Cox proportional hazard models to obtain hazard ratios (HRs) and their 95% confidence intervals (CIs). Kaplan–Meier curves were used to illustrate the relationship between each PC and survival. The predictive value of the PCs on the rate of functional decline, defined by ALSFRS-R scores after study participation, was examined using linear mixed-effects models. Fixed effects were the time since first assessment (in months), PC magnitude and an interaction between PC magnitude and time. We fitted a random intercept and slope for time for each individual. Significance testing was based on the likelihood ratio test. To study the additional predictive value of the PCs, we added the ENCALS risk score as a predictor for the Cox proportional hazards models and the linear mixed-effects models. For the latter, we incorporated an interaction term between time and the ENCALS risk score. To address the large series of tests, *P*-values corresponding to the group effect were adjusted with Benjamini–Hochberg’s false-detection rate procedure.^33^ An adjusted *P <* 0.05 was considered statistically significant. All statistical analyses were performed in R (http://cran.r-project.org).

## Results

### Demographics and clinical characteristics

We recruited a total of 161 ALS patients, whose characteristics are presented in Table 1. To summarize, age and sex were comparable between patients and controls. Patients were examined after a median disease duration of 11 months in early disease stages (79% in Kings’ stage 1–2; median ALSFRS-R = 41). The median (interquartile range, IQR) ΔFRS at visit was 0.6 (0.4–1.1) points/month, followed by an ALSFRS-R slope after study participation of 0.8 (0.6–0.9) points/month (available for 86 patients; median scores per individual = 4). At the time of writing, 80 of 161 patients (50%) reached the composite survival end point after a median (IQR) time of 33 (22–44) months.

**Table 1:** Characteristics of the patients with amyotrophic lateral sclerosis and healthy controls

Characteristic	ALS *n* = 161	Control *n* = 50	*P*-value
**Demographics**			
Age, years	64 (58–70)	65 (55–69)	0.4
Sex, male/female	100/61	27/23	0.3
Symptom duration since onset, months	11 (6–17)	–	–
Site of onset, spinal/bulbar	119/42	–	–
**Disease stage**			
King’s stage, 1/2/3/4a/4b	76/51/31/1/2	–	–
ALSFRS-R score	41 (38–44)	–	–
FMF score	10 (9–12)	–	–
MUNE	45 (24–74)	78 (68–96)	<0.001
**Disease progression rate**			
ΔFRS	0.6 (0.4–1.1)	–	–
ENCALS risk profile	−4.2 (–4.9 to −3.6)	–	–

Data are presented as the median (25th–75th percentile) or as group counts. ALS = amyotrophic lateral sclerosis; ALSFRS-R = ALS functional rating scale (revised); ENCALS risk profile = score obtained from the ENCALS prediction model^22^; FMF = fine motor function (items 4–6 of ALSFRS-R); ΔFRS = decline in ALSFRS-R score per month; King’s stage = King’s clinical staging system; MUNE = motor unit number estimate.

### Nerve excitability changes in ALS patients

**Figure 2:**
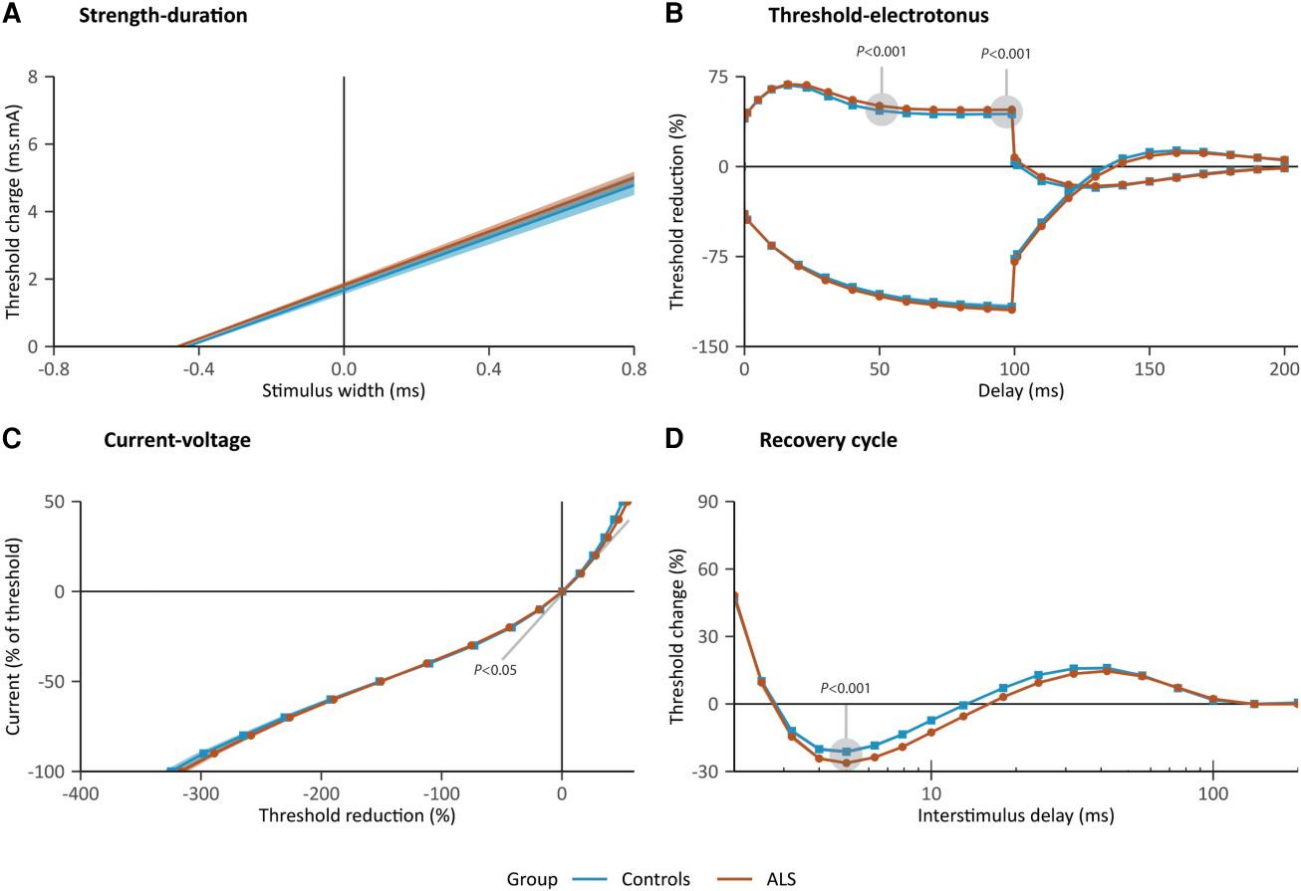
**Averaged excitability recordings of amyotrophic lateral sclerosis patients (*n* = 161, orange) and controls (*n* = 50, blue).** Plots represent the standard tests from the TRONDNF protocol, with: (**A**) the strength–duration test; (**B**) the threshold electrotonus (only 40% hyperpolarizing and depolarizing conditioning currents are shown for clarity), with elevated values in patients between 40 and 60 ms and between 90 and 100 ms during 40% depolarization (TEd_40–60ms_ and TEd_90–100ms_); (**C**) the current–voltage test, with less steep slopes at zero conditioning in patients (resting *I*/*V* slope); and (**D**) the recovery cycle, with decreased superexcitability in patients. Shading around the lines represents the standard error; note that shading is present in **B** and **D**, albeit very narrow. *P­*-values were obtained from a linear regression model with age and sex as covariates, adjusted by false-detection rate correction.

Differences between standard excitability measures of patients and controls were identified during the threshold electrotonus, current–voltage relationship and the recovery cycle (Fig. 2). A summary of the standard excitability measures in patients and controls is provided in Table 2. Briefly, patients exhibited distinctly higher TEd_40–60ms_ (*t =* 5.4, *P* < 0.001), higher TEd_90–100ms_ (*t =* 4.7, *P* < 0.001) and increased superexcitability (*t =* 4.9, *P* < 0.001). Minor differences between patients and controls were also identified at TEd_Peak_, in the S2 accommodation and in the resting *I*/*V* slope. No significant relationships were identified between the standard excitability measures and site of symptom onset (spinal versus bulbar) or the disease duration.

**Table 2:** Summary of standard nerve excitability measures and their corresponding PCs

Characteristic	ALS *n* = 161	Controls *n* = 50	Strongest correlation with:	*r* (95% CI)
**Strength–duration**				
SDTC (µs)	458 ± 8	433 ± 8	PC3	0.43 (0.29, 0.55)
Rheobase (mA)	4.0 ± 0.1	3.9 ± 0.2	PC3	−0.39 (−0.51, −0.25)
**Threshold electrotonus**				
TEd_Peak_ (%)	66.9 ± 0.4*	65.3 ± 0.5	PC1	0.67 (0.57, 0.75)
TEd_40–60ms_ (%)	51.1 ± 0.4***	47.3 ± 0.5	PC1	0.67 (0.58, 0.75)
TE­d_90–100ms_ (%)	47.2 ± 0.4***	43.8 ± 0.5	PC1	0.72 (0.64, 0.79)
TEd_Undershoot_ (%)	−16.2 ± 0.4	−17.2 ± 0.3	PC2	0.89 (0.86, 0.92)
S2 accommodation (%)	19.9 ± 0.4*	21.5 ± 0.4	PC2	−0.73 (−0.80, −0.65)
TEh_90–100ms_ (%)	−119.1 ± 1.6	−116.4 ± 2.1	PC1	−0.78 [−0.83, −0.70)
TEh_Overshoot_ (%)	13.6 ± 0.4	13.9 ± 0.5	PC2	−0.66 (−0.74, −0.57)
Fanning (%)	166.3 ± 1.8	160.2 ± 2.4	PC1	0.86 (0.82, 0.90)
**Current–voltage**				
Resting *I*/*V* slope (10^−2^)	58.6 ± 0.6*	61.7 ± 1.1	PC1	−0.78 (−0.83, −0.71)
Minimum *I*/*V* slope (10^−2^)	25.5 ± 0.4	24.5 ± 0.6	PC3	0.59 (0.48, 0.68)
Hyperpolarizing *I*/*V* slope (10^−2^)	35.3 ± 0.5	34.0 ± 0.7	PC3	0.14 (−0.01, 0.29)
**Recovery cycle**				
Refractoriness (%)	49.4 ± 2.7	47.4 ± 2.3	PC4	0.55 (0.43, 0.65)
RRP (µs)	2716 ± 29	2794 ± 360	PC4	0.54 (0.43–0.64)
Superexcitability (%)	25.1 ± 0.6***	20.1 ± 0.8	PC1	0.71 (0.62, 0.78)
Subexcitability (%)	14.1 ± 0.4	15.3 ± 0.6	PC2	−0.51 (−0.62, −0.38)
**Excitability patterns**				
PC1	5.7 ± 0.7***	0 ± 1.0	–	–
PC2	2.1 ± 0.6*	0 ± 0.6	–	–
PC3	1.7 ± 0.4*	0 ± 0.4	–	–
PC4	0.8 ± 0.4	0 ± 0.5	–	–

Data are presented as the mean ± standard error (of the mean or mean difference). Values within parentheses indicate 95% confidence intervals (CI). PC = principal component; *r* = Pearson’s correlation coefficient with the given PC.

*P < 0.05; **P < 0.01; ***P < 0.001.

### Unique patterns of nerve excitability changes

**Figure 3:**
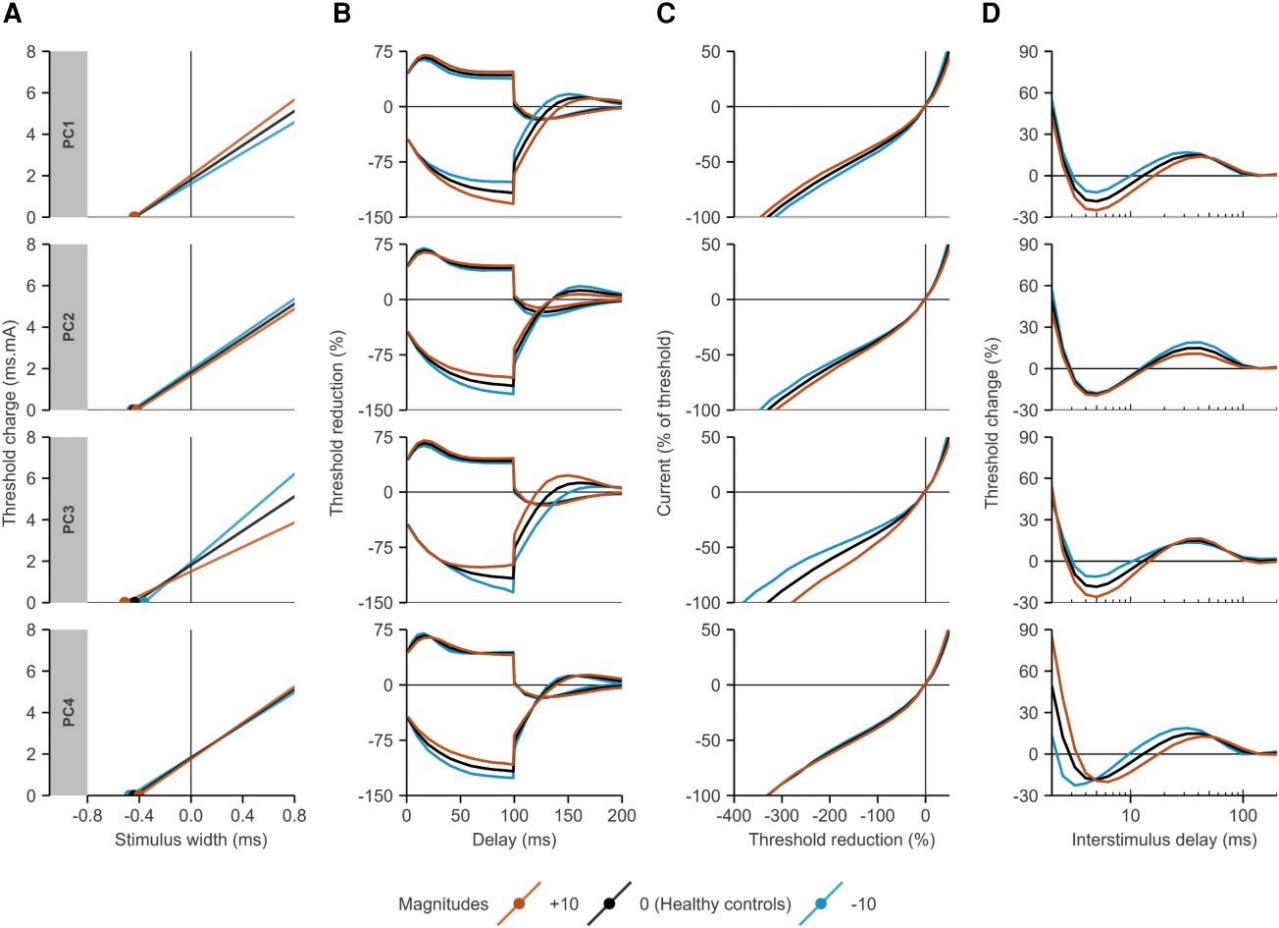
**Unique patterns of excitability changes in patients with ALS.** (**A**) Strength–duration test. (**B**) Threshold electrotonus. (**C**) Current–voltage test. (**D**) Recovery cycle test. Red lines and blue lines indicate the effects of increasing or decreasing PC magnitudes by 10, respectively, with a PC magnitude of zero (black) representing the average recording in healthy controls. PC = principal component.

We retrieved four unique excitability patterns based on PCA, which together accounted for 73% of the total variance in the measures (PC 1–4: 36%, 18%, 10% and 9%). The corresponding patterns are presented in Fig. 3 at two different magnitudes (−10 and +10) for illustrative purposes. These four components provided the best description of the available information in the recordings and were found to be stable in our internal validation (Supplementary material). The magnitudes of the PCs in ALS patients and non-neurological controls are presented in Table 2. In the full cohort, patients exhibited larger magnitudes in the first three PCs. Additionally, we calculated the correlation coefficients between all standard excitability measures and PCs; we present the PC to which the standard measures were most strongly correlated in Table 2. Unsurprisingly, the first pattern, described by PC1, was strongly correlated with the most distinctive standard excitability measures for ALS patients (TEd_40–60ms_, TEd_90–100ms_ and superexcitability; all *P* < 0.001; Table 2). The pattern described by PC2 mainly captured variance in the accommodation phase of the depolarizing threshold electrotonus and the subexcitable phase of the recovery cycle, whereas the pattern described by PC3 captured effects throughout the four excitability tests, most distinctively in the strength–duration test. Lastly, the pattern described by PC4 mainly captured the temporal profile of the recovery cycle.

### Association between excitability patterns and clinical measures

**Figure 4:**
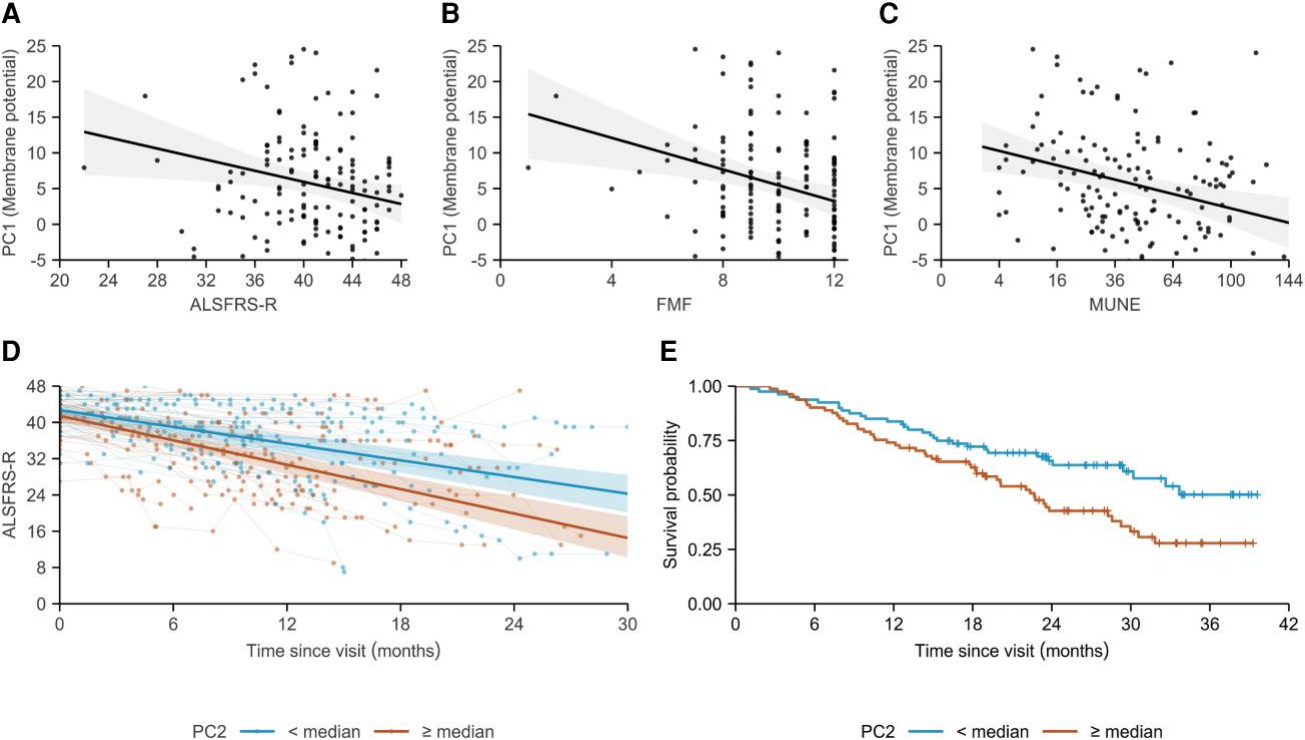
**Disease-specific patterns of excitability changes relate to disease stage or progression rate.** (**A**) Revised ALS functional rating scores (ALSFRS-R). (**B**) Fine motor function subdomain scores (FMF; items 4–6 of ALSFRS-R). (**C**) Motor unit number estimates (MUNE) with respect to the magnitude of PC1 in all patients (black). (**D**) Follow-up ALSFRS-R scores after visit (*n* = 86), based on the magnitude of PC2 stratified by the cohort median, with those below the median (blue) progressing 1.5 times more slowly than those above (orange; *P* = 0.005, results from linear mixed-effects model as described in the statistical analysis section). (**E**) Kaplan–Meier curves stratified in a similar manner to those in **D**. ALS = amyotrophic lateral sclerosis; PC = principal component.

The identified excitability patterns showed no association with the patients’ sites of symptom onset (spinal versus bulbar) nor with their disease durations. However, we found that patients in advanced disease stages had more aberrant PC1 magnitudes [coefficient *±* standard error (SE): ALSFRS-R = −0.39 *±* 0.16, *t* = −2.5, *P* = 0.015; FMF = −1.11 *±* 0.35, *t* = −3.1, *P* = 0.002; MUNE = −1.01 *±* 0.30, *t =* −3.3, *P =* 0.001; Fig. 4A–C]. To test whether these results were driven by a small number of patients with severely reduced clinical scores (left tails of Fig. 4A and B), we repeated the analyses while omitting patients with ALSFRS-R or FMF scores within the lower 5th percentile. This omission had minimal effect on the identified relationships; *P-*values were marginally increased but remained below the significance threshold.

Measures quantifying the disease progression rate were related only to the magnitude of PC2 [coefficient *±* SE: log(ΔFRS) = 3.26 ± 0.97, *t =* 3.4, *P* < 0.001; ENCALS risk score = 2.18 ± 0.84, *t =* 2.6, *P* = 0.010]. Of note, the identified relationship between ΔFRS and PC2 was more pronounced in patients with lower MUNE (*P* = 0.012). Higher PC2 values were also predictive of steeper ALSFRS-R slopes after the study visit (coefficient *±* SE *=* −0.26 ± 0.09, *P =* 0.005). Based on the model coefficients, average rates of decline of patients below or above the median of PC2 were −7.4 and −10.8 ALSFRS-R points/year, respectively (1.5 times faster; Fig. 4D). PC2 was a strong predictor for the ALSFRS-R slope even when the ENCALS risk score was incorporated in the model as a predictor (coefficient *±* SE: PC2 = −0.23 ± 0.09, *P* = 0.010; ENCALS risk score *=* −1.66 ± 0.57, *P* = 0.004). The relationship between PC2 and survival since study participation was significant [HR (95% CI) = 1.03 (1.01–1.07), *P* = 0.022]. We illustrate the survival of patients with PC2 above or below the group median in Fig. 4E. Upon addition of the ENCALS risk score into the model, the effect of PC2 as a predictor was reduced [HR (95% CI) = 1.03 (1.00–1.06), *P* = 0.055]. This finding suggests that the added value of nerve excitability measures to the current prediction model might be limited. No associations were identified between PC3 or PC4 and any of the clinical measures examined.

### Biophysical basis of the excitability patterns

**Figure 5:**
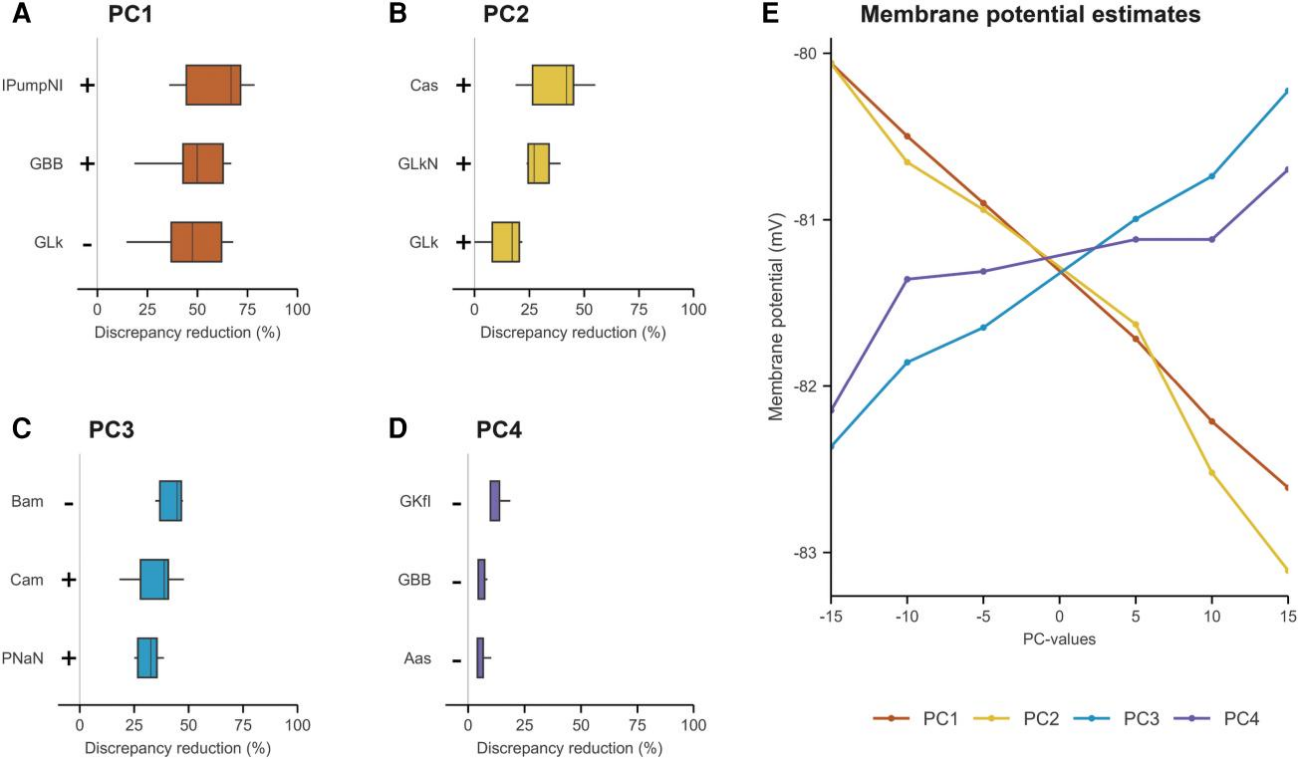
**Discrepancy reduction obtained by the three major determinant membrane properties of each principal component.** (**A**–**D**) Box plots in each panel represent the discrepancy reductions per parameter from an optimized nerve model that simulated the PC patterns at magnitudes ranging from −15 to 15 in steps of 5 (*n* = 7 points). Minus signs indicate that a decrease in PC magnitude corresponded to a reduction in that parameter, and vice versa for plus signs. (**E**) The estimated (nodal) resting membrane potential at the examined PC values, corresponding to a change in the best parameters (**A**–**D**, *top rows*). Aas = activation rate of slow fast potassium channels; Bam = voltage of half-activation of sodium channels; Cas, Cam = voltage activation slope factor of slow potassium channels and sodium channels; GBB = Barrett–Barrett conductance; GLk-GLkN = inter- and nodal leak-channel conductance; GKfI = internodal fast potassium-channel conductance; IPumpNI = (inter-)nodal Na^+^/K^+^-pump currents; PNaN = permeability of sodium channels.

The three most likely biophysical determinants of each PC pattern, the corresponding discrepancy reductions and the estimated resting membrane potentials at each PC value are illustrated in Fig. 5. The pattern described by PC1 was best modelled by voltage-independent properties, most notably the membrane potential (modulated by Na^+^/K^+^-pump currents). Higher PC1 values corresponded to more membrane hyperpolarization (i.e. larger Na^+^/K^+^­-pump currents). Given that PC1 was increased in more severely affected patients, this finding suggests that motor axons are increasingly hyperpolarized in more severely affected muscles. The pattern in PC2 was best described by the gating kinetics of slow potassium channels. Specifically, an increase in PC2 corresponded to a decrease in the steepness of the voltage-dependent activation slope of slow K^+^ channels, resulting in relatively increased potassium currents and a net hyperpolarization effect on the membrane potential. Combined with the observed associations between PC2 and measures of disease progression rate, these findings indicate that less steep voltage-dependent activation slope of slow K^+^ channels might relate to faster functional decline. The pattern of PC3 was best described by parameters associated with gating kinetics and permeability of sodium channels. The percentage of persistent sodium channels was also an important determinant of the excitability pattern of PC3, albeit to a lesser extent than the permeability (1.3% less discrepancy reduction). Altogether, larger values of PC3 corresponded to larger sodium currents and, consequently, a net depolarization of the membrane potential. The pattern of PC4, associated with changes in the refractory period, could not be reproduced adequately by the nerve model used. Overall, these findings indicate that the magnitudes of PC1–PC3 could potentially serve as proxy measures for specific membrane properties and mechanisms.

### Identified patterns captured by composite excitability measures

We found that a subset of only 10 distinct standard excitability measures were required to adequately quantify the four identified excitability patterns. The corresponding composite measures, each of which describes one excitability pattern, can now be calculated for an individual patient using the following equations:


(1) $$\mathrm{Composite}\;A\;(\mathrm{PC}1,\;\mathrm{membrane}\;\mathrm{potential})=\;-3.92\times \mathrm{TE}h_{90-100\mathrm{ms}}+2.57\times \mathrm{TE}d_{40-60\mathrm{ms}}+1.91\times \mathrm{Superexcitability}-0.26\times \mathrm{TE}h_{\mathrm{overshoot}}-0.07\times S2$$

(2) $$\;\mathrm{Composite}\;B\;(\mathrm{PC}2,\;\mathrm{slow}\;K^{+}\;\mathrm{gating})=3.00\times \mathrm{TE}d_{\mathrm{undershoot}}+2.06\times \mathrm{TE}h_{90-100\mathrm{ms}}-1.15\times {\mathrm{TEh}}_{\;\mathrm{overshoot}}+1.02\times \mathrm{TE}d_{\mathrm{peak}}-0.20\times \mathrm{Refractoriness}$$

(3) $$\mathrm{Composite}\;C\;(\mathrm{PC}3,\;Na^{+\;}\mathrm{gating})=2.00\times \mathrm{TE}h_{90-100\mathrm{ms}}+1.80\times \mathrm{Superexcitability}+1.71\times \mathrm{TE}h_{\mathrm{overshoot}}+\;0.79\times \mathrm{SDTC}-0.63\times \mathrm{Subexcitability}$$

(4) $$\mathrm{Composite}\;D\;(\mathrm{PC}4,\;\mathrm{refractory}\;\mathrm{properties})=1.77\times \mathrm{Refractoriness}-1.53\times \mathrm{SDTC}+0.88\times {\mathrm{TEd}}_{\;40-60\mathrm{ms}}+0.43\times S2+0.37\times \mathrm{Superexcitability}$$

The correlation coefficients between the composites and the excitability patterns were 0.96, 0.96, 0.88 and 0.74 for composites A–D, respectively. The measures in the formulae above were standardized with respect to controls by subtracting the mean, then dividing by the standard deviation from controls, using the data presented in Table 2. In practice, these composite measures might serve as rapid proxy measures for the identified biophysical mechanisms in individual patients.

## Discussion

In this study, we revisited nerve excitability changes in patients with ALS using a new analysis approach, demonstrating that excitability changes in ALS patients are characterized by four distinct patterns, each originating from changes in different membrane properties. The magnitudes of these patterns, obtained directly from recordings of individual patients, can, therefore, provide insights into the underlying membrane properties. Most notably, we showed that excitability changes related to altered gating kinetics of slow potassium channels were associated with, and predictive of, the progression rates of patients. Based on our findings, we propose a set of composite measures that can be derived quickly, for practical implementation in clinical practice and trials.

### Axonal hyperpolarization in increasingly affected muscles

Overall, the nerve excitability measures in our cohort were comparable with previous studies, showing increases in TEd_90–100_ and superexcitability as most distinctive measures for ALS patients.^9,34-38^ These combined excitability changes were captured well by the excitability pattern that accounted for most of the variance observed in patients, e.g. PC1. We found that this excitability pattern was more aberrant in patients with lower clinical scores and lower MUNE in the examined muscle. However, we observed marked between-subject variability in these relationships that could be attributed to the study design, single nerve–muscle evaluation or clinical variability in the patient population. Nonetheless, these relationships were in line with those identified in a previous longitudinal study, in which progressive increases in TEd_90–100_ and superexcitability were observed over a 12-week period.^39^ A study of single motor units also found a comparable pattern of excitability changes in motor units from more severely affected parent muscles, in comparison to single motor units from moderately affected pools and healthy motor units.^15^ Based on our modelling results, this progressive change in excitability was consistent with a progressively hyperpolarized membrane potential. In light of these findings, we hypothesize that these well-documented excitability changes originate from an increasingly resistant set of surviving axons that is relatively hyperpolarized with respect to the deceased axons.

### Altered slow potassium-channel gating kinetics are related to the rate of disease progression

Our data suggest that the second disease-specific pattern of excitability changes, captured by PC2, was related to various measures of the disease progression rate. This pattern was best modelled by changes in the gating kinetics of slow potassium channels. Only a limited number of studies have examined the relationship between *in vivo* excitability measures and the progression rate of ALS.^38,40^ Neither of these studies identified any of the measures related to PC2 as relevant predictors. Several differences should be acknowledged, including inclusion at an early disease stage during the diagnostic process, resulting in recordings from exclusively treatment-naïve patients, and differences in the frequency of each of the survival end points (e.g. tracheostomy, non-invasive ventilation or death). Furthermore, differences in results from these previous studies and ours might have originated from the warming procedure in our study, given that temperature increases have a major effect on slow potassium-channel gating kinetics.^41^ It is possible that temperature-induced variability was smaller in our study, thereby augmenting the statistical power to detect associations with clinical properties such as the disease progression rate. Contrary to the favoured hypothesis that hyperexcitability might promote neurodegeneration, recent preclinical studies have suggested that motor neuron hyperexcitability is an unlikely driver of excitotoxicity,^42-44^ with some even showing that hyperexcitable motor neurons are more resistant to degeneration.^45^ The changes we identified in potassium-channel gating kinetics would, likewise, drive the resting membrane potential to hyperpolarization rather than depolarization. In this context, it is crucial to distinguish between *in vivo* excitability studies, targeting the distal part of the axon, and preclinical cellular studies, focusing on excitability in the cell body. Their combined findings, however, might offer relevant translational insights into the effects of altered membrane excitability on motor neuronal survival. Although somewhat semantic, one could argue that ion channels that are opened further at the resting membrane potential than normal exhibit a type of ion-channel hyperexcitability, because they allow an inadvertent flow of current that would otherwise not occur. Overall, our study is one of few to provide *in vivo* insights into the relationship between the progression rates of ALS patients and nerve excitability. In light of the interaction between axon loss (lower MUNE) and the magnitude of PC2, however, longitudinal assessment is warranted to determine whether this observed excitability pattern reflects a transient property, secondary to the main degenerative process, or whether it represents a primary driving mechanism.

### Clinical characteristics of patients are not related to sodium-channel properties

We found that the excitability pattern captured by PC3 was determined predominantly by sodium-associated properties. Traditionally, the SDTC has been considered an important determinant of changes in persistent sodium currents, which was frequently found to be elevated in ALS.^5,9,36,37,39,46^ In some studies, however, differences in the SDTC of patients and controls were difficult to demonstrate,^47^ as was also the case in our results. Nevertheless, the magnitude of PC3 did differ significantly between patients and controls, providing further supporting evidence for elevated sodium currents in ALS. Potentially, changes in sodium currents might precede changes in potassium currents, promoting an initial state of hyperexcitability and axonal membrane instability that manifests clinically as fasciculations.^48^ Surprisingly, we were not able to reproduce the results from two studies that examined the relationship between excitability and disease progression rates that identified SDTC and consequently persistent sodium currents as an important predictor.^38,40^ As addressed in the previous section, however, there were several methodological differences between these two studies and our study. In the absence of further associations with any of the examined clinical characteristics, the nature of altered sodium-channel properties in ALS, whether reflecting a transient feature of the disease pathophysiology,^42^ a compensatory mechanism^45^ or expression of an early step in the proposed multi-step process to developing ALS,^49^ remains to be elucidated in longitudinal studies.

### Clinical relevance

We have demonstrated that ALS is characterized by four unique patterns of excitability changes that reflect important abnormalities in the underlying biophysical properties of the motor axons of patients, including membrane potential, slow potassium-channel gating kinetics, sodium currents and refractory properties. Importantly, these underlying properties can be studied directly and efficiently from excitability recordings of individual patients using the pattern magnitudes. One of these patterns was more aberrant in patients at more advanced disease stages, whereas another was more aberrant in patients with faster disease progression rates. This distinction is important when considering the application of this neurophysiological technique in clinical practice or clinical trials. Pharmacological modulation of an excitability pattern that relates exclusively to the disease stage, e.g. PC1, might have limited value in clinical trials, because normalization of this pattern might obscure ongoing progression, rather than reflect a halt of the disease. In contrast, the identification of an excitability pattern that was strongly related to the current progression rate of the disease is highly informative, especially given that it could be established by a single observation. These measures might be valuable to facilitate rapid characterization of differences in individual responses to new treatments, although more work is needed to validate (externally) the biophysical origin and temporal attributes underpinning these excitability changes, in addition to their association with clinical characteristics.

To advance the application of excitability tests further in clinical practice, we propose a new set of biophysically informed composite excitability measures that capture the PC patterns. We developed practical composites such that these can be calculated directly from a subset of established standard excitability measures to facilitate wider implementation by other researchers and clinicians. This subset of measures, importantly, includes the excitability measures that were shown to have high diagnostic accuracy in the same single nerve–muscle combination, irrespective of the site of onset or the presence of axon loss.^23^ As such, physicians could opt to obtain only this subset of excitability measures to help with diagnosis, to provide baseline information for clinical trials or to follow the development of excitability changes longitudinally. This would greatly reduce the number of measures required to be interpreted in analyses, while retaining the underlying biophysical information in order to provide crucial mechanistic insights.

### Limitations

The main limitation of this study is that we examined only one nerve–muscle combination in a cross-sectional manner. Evaluation of longitudinal trajectories of excitability changes and multiple nerve–muscle combinations could provide further valuable insights into the clinical heterogeneity. However, the observed relationships between disease stage and progression rate were significant at various levels, including that of the full body, cervical region and the muscle itself. Therefore, in the absence of a relationship between the site of onset or disease duration, we think that our results are representative for this diverse population. Results from our survival analyses might have been incurred a minor bias attributable to the relatively low number of deceased patients, owing to the prospective nature of the study. Future work should address the added predictive value of nerve excitability measures to established prognostic models for ALS in larger samples, by refitting all variables in the ENCALS prediction model. We believe, however, that the strong predictive value of nerve excitability measures on the rate of decline of ALSFRS-R scores supports the observed trends in our survival analyses. PCA evaluates linear relationships between data, although more complex associations might exist. Further improvements in this approach might involve the implementation of non-linear techniques, such as neural network-based auto-encoders or non-linear PCA, potentially to retrieve even more biophysically informed measures directly from these neurophysiological recordings. The incorporation of additional predictors of peripheral excitability, such as serum levels of potassium,^31^ in the standardization procedure could further improve the ascription of variance to pathophysiological changes. The modelling itself relied on a model of a single node–internode combination, with voltage-gated ion channels described as electrical components rather than complex protein microstructures that cannot studied within this electrophysiological framework. This single-axon model could, additionally, not account for potential changes distal to the stimulus site that are observed mainly in the refractory period,^50^ as captured by PC4. Supplementing the excitability data with tests not included in the current TRONDNF protocol, such as latent addition, could reveal additional excitability patterns or strengthen those identified in this study. However, at present no other standard protocol is available for testing excitability and is, therefore, more challenging to implement. Finally, nerve excitability testing provides valuable insights into subsets of motor neurons by tracking a predefined target CMAP (typically 40% of the maximum CMAP). Excitability studies have shown variable motor neuron properties when studying different target amplitudes.^51^ This physiological characteristic should be kept in mind when using nerve excitability tests as a tool to translate between preclinical and clinical findings.

## Conclusion

Our findings have provided several new insights into the relationships between clinical characteristics of ALS patients and nerve excitability measures. Longitudinal examinations will be required to characterize the temporal attributes of the identified patterns of excitability changes better. Nevertheless, we provide new *in vivo* evidence that altered gating kinetics of slow potassium channels are related to the disease progression rate. This electrophysiological signature can now be obtained efficiently from an excitability recording of an individual patient using the proposed composite excitability measures and could be valuable as a biomarker for future trials. In summary, we believe that our findings, our approach to the analysis and the new composite measures can be used to leverage this neurophysiological technique optimally to the benefit of clinical practice and the testing of new drugs in patients with ALS.

## Supplementary Material

awae131_Supplementary_Data

## Data Availability

The study data supporting the findings are available on reasonable request via the corresponding author.

## References

[1] van Es MA, Hardiman O, Chio A, et al Amyotrophic lateral sclerosis. Lancet. 2017;390:2084–2098. 28552366 10.1016/S0140-6736(17)31287-4

[2] Park SB, Kiernan MC, Vucic S. Axonal excitability in amyotrophic lateral sclerosis: Axonal excitability in ALS. Neurotherapeutics. 2017;14:78–90. 27878516 10.1007/s13311-016-0492-9 PMC5233634

[3] Bae JS, Simon NG, Menon P, Vucic S, Kiernan MC. The puzzling case of hyperexcitability in amyotrophic lateral sclerosis. J Clin Neurol. 2013;9:65–74. 23626643 10.3988/jcn.2013.9.2.65 PMC3633193

[4] Gunes ZI, Kan VWY, Ye XQ, Liebscher S. Exciting complexity: The role of motor circuit elements in ALS pathophysiology. Front Neurosci. 2020;14:573. 32625051 10.3389/fnins.2020.00573 PMC7311855

[5] Iwai Y, Shibuya K, Misawa S, et al Axonal dysfunction precedes motor neuronal death in amyotrophic lateral sclerosis. PLoS One. 2016;11:e0158596. 27383069 10.1371/journal.pone.0158596 PMC4934877

[6] Kovalchuk MO, Heuberger J, Sleutjes B, et al Acute effects of riluzole and retigabine on axonal excitability in patients with amyotrophic lateral sclerosis: A randomized, double-blind, placebo-controlled, crossover trial. Clin Pharmacol Ther. 2018;104:1136–1145. 29672831 10.1002/cpt.1096

[7] Wainger BJ, Macklin EA, Vucic S, et al Effect of ezogabine on cortical and spinal motor neuron excitability in amyotrophic lateral sclerosis: A randomized clinical trial. JAMA Neurol. 2021;78:186–196. 33226425 10.1001/jamaneurol.2020.4300 PMC7684515

[8] Park SB, Vucic S, Cheah BC, et al Flecainide in amyotrophic lateral sclerosis as a neuroprotective strategy (FANS): A randomized placebo-controlled trial. EBioMedicine. 2015;2:1916–1922. 26844270 10.1016/j.ebiom.2015.11.022 PMC4703720

[9] Vucic S, Lin CS, Cheah BC, et al Riluzole exerts central and peripheral modulating effects in amyotrophic lateral sclerosis. Brain. 2013;136(Pt 5):1361–1370. 23616585 10.1093/brain/awt085

[10] Geevasinga N, Menon P, Ng K, et al Riluzole exerts transient modulating effects on cortical and axonal hyperexcitability in ALS. Amyotroph Lateral Scler Frontotemporal Degener. 2016;17(7–8):580–588. 27249331 10.1080/21678421.2016.1188961

[11] Bostock H, Cikurel K, Burke D. Threshold tracking techniques in the study of human peripheral nerve. Muscle Nerve. 1998;21:137–158. 9466589 10.1002/(sici)1097-4598(199802)21:2<137::aid-mus1>3.0.co;2-c

[12] Kiernan MC, Bostock H, Park SB, et al Measurement of axonal excitability: Consensus guidelines. Clin Neurophysiol. 2020;131:308–323. 31471200 10.1016/j.clinph.2019.07.023

[13] Kiernan MC, Isbister GK, Lin C S-Y, Burke D, Bostock H. Acute tetrodotoxin-induced neurotoxicity after ingestion of puffer fish. Ann Neurol. 2005;57:339–348. 15732107 10.1002/ana.20395

[14] Howells J, Trevillion L, Bostock H, Burke D. The voltage dependence of *I*_h_ in human myelinated axons. J Physiol. 2012;590:1625–1640. 22310314 10.1113/jphysiol.2011.225573 PMC3413487

[15] Howells J, Matamala JM, Park SB, et al *In vivo* evidence for reduced ion channel expression in motor axons of patients with amyotrophic lateral sclerosis. J Physiol. 2018;596:5379–5396. 30175403 10.1113/JP276624 PMC6235931

[16] Sleutjes BTHM, Stikvoort García DJL, Kovalchuk MO, et al Acute retigabine-induced effects on myelinated motor axons in amyotrophic lateral sclerosis. Pharmacol Res Perspect. 2022;10:e00983. 35881020 10.1002/prp2.983 PMC9318643

[17] Kiernan M, Bostock H. Effects of membrane polarization and ischaemia on the excitability properties of human motor axons. Brain. 2000;123:2542–2551. 11099455 10.1093/brain/123.12.2542

[18] Huisman MHB, De Jong SW, Van Doormaal PTC, et al Population based epidemiology of amyotrophic lateral sclerosis using capture–recapture methodology. J Neurol Neurosurg Psychiatry. 2011;82:1165–1170. 21622937 10.1136/jnnp.2011.244939

[19] Kovalchuk MO, Franssen H, Van Schelven LJ, Sleutjes BTHM. Comparing excitability at 37°C versus at 20°C: Differences between motor and sensory axons. Muscle Nerve. 2018;57:574–580. 28877547 10.1002/mus.25960

[20] Kovalchuk MO, Franssen H, van den Berg LH, van Schelven LJ, Sleutjes B. Excitability of motor and sensory axons in multifocal motor neuropathy. Clin Neurophysiol. 2020;131:2641–2650. 32947198 10.1016/j.clinph.2020.08.004

[21] Cedarbaum JM, Stambler N, Malta E, et al The ALSFRS-R: A revised ALS functional rating scale that incorporates assessments of respiratory function. J Neurol Sci. 1999;169(1–2):13–21. 10540002 10.1016/s0022-510x(99)00210-5

[22] Westeneng HJ, Debray TPA, Visser AE, et al Prognosis for patients with amyotrophic lateral sclerosis: Development and validation of a personalised prediction model. Lancet Neurol. 2018;17:423–433. 29598923 10.1016/S1474-4422(18)30089-9

[23] Stikvoort García DJL, Sleutjes B, van Schelven LJ, Goedee HS, van den Berg LH. Diagnostic accuracy of nerve excitability and compound muscle action potential scan derived biomarkers in amyotrophic lateral sclerosis. Eur J Neurol. 2023;30:3068–3078. 37354059 10.1111/ene.15954

[24] Kovalchuk MO, Franssen H, Scheijmans FEV, Van Schelven LJ, Van Den Berg LH, Sleutjes BTHM. Warming nerves for excitability testing. Muscle Nerve. 2019;60:279–285. 31241195 10.1002/mus.26621

[25] Kiernan MC, Burke D, Andersen KV, Bostock H. Multiple measures of axonal excitability: A new approach in clinical testing. Muscle Nerve. 2000;23:399–409. 10679717 10.1002/(sici)1097-4598(200003)23:3<399::aid-mus12>3.0.co;2-g

[26] Sleutjes BTHM, Montfoort I, Maathuis EM, et al CMAP scan discontinuities: Automated detection and relation to motor unit loss. Clin Neurophysiol. 2014;125:388–395. 23993681 10.1016/j.clinph.2013.07.016

[27] Bostock H . Estimating motor unit numbers from a CMAP scan. Muscle Nerve. 2016;53:889–896. 26479267 10.1002/mus.24945

[28] Jacobsen AB, Bostock H, Fuglsang-Frederiksen A, et al Reproducibility, and sensitivity to motor unit loss in amyotrophic lateral sclerosis, of a novel MUNE method: MScanFit MUNE. Clin Neurophysiol. 2017;128:1380–1388. 28461135 10.1016/j.clinph.2017.03.045

[29] Casanova I, Diaz A, Pinto S, De Carvalho M. Motor excitability measurements: The influence of gender, body mass index, age and temperature in healthy controls. Neurophysiol Clin. 2014;44:213–218. 24930943 10.1016/j.neucli.2014.03.002

[30] Bae JS, Sawai S, Misawa S, et al Effects of age on excitability properties in human motor axons. Clin Neurophysiol. 2008;119:2282–2286. 18760964 10.1016/j.clinph.2008.07.005

[31] Boërio D, Bostock H, Spescha R, Z’Graggen WJ. Potassium and the excitability properties of normal human motor axons *in vivo*. PLoS One. 2014;9:e98262. 24893161 10.1371/journal.pone.0098262 PMC4043986

[32] Tomlinson SE, Tan SV, Burke D, et al *In vivo* impact of presynaptic calcium channel dysfunction on motor axons in episodic ataxia type 2. Brain. 2016;139:380–391. 26912519 10.1093/brain/awv380 PMC4795516

[33] Benjamini Y, Hochberg Y. Controlling the false discovery rate: A practical and powerful approach to multiple testing. J R Stat Soc. 1995;57:289–300.

[34] Kanai K, Kuwabara S, Misawa S, et al Altered axonal excitability properties in amyotrophic lateral sclerosis: Impaired potassium channel function related to disease stage. Brain. 2006; 129(Pt 4):953–962. 16467388 10.1093/brain/awl024

[35] Noto YI, Kanai K, Misawa S, et al Distal motor axonal dysfunction in amyotrophic lateral sclerosis. J Neurol Sci. 2011;302(1–2):58–62. 21195434 10.1016/j.jns.2010.11.025

[36] Shibuya K, Misawa S, Nasu S, et al Split hand syndrome in amyotrophic lateral sclerosis: Different excitability changes in the thenar and hypothenar motor axons. J Neurol Neurosurg Psychiatry. 2013;84:969–972. 23467416 10.1136/jnnp-2012-304109

[37] Geevasinga N, Menon P, Howells J, Nicholson GA, Kiernan MC, Vucic S. Axonal ion channel dysfunction in c9orf72 familial amyotrophic lateral sclerosis. JAMA Neurol. 2015;72:49–57. 25384182 10.1001/jamaneurol.2014.2940

[38] Shibuya K, Misawa S, Kimura H, et al Increased motor axonal persistent sodium currents predict rapid functional declines in amyotrophic lateral sclerosis. Neurol Clin Neurosci. 2016;4:108–111.

[39] Cheah BC, Lin CS, Park SB, Vucic S, Krishnan AV, Kiernan MC. Progressive axonal dysfunction and clinical impairment in amyotrophic lateral sclerosis. Clin Neurophysiol. 2012;123:2460–2467. 22921484 10.1016/j.clinph.2012.06.020

[40] Kanai K, Shibuya K, Sato Y, et al Motor axonal excitability properties are strong predictors for survival in amyotrophic lateral sclerosis. J Neurol Neurosurg Psychiatry. 2012;83:734–738. 22566594 10.1136/jnnp-2011-301782

[41] Howells J, Czesnik D, Trevillion L. Excitability and the safety margin in human axons during hyperthermia. J Physiol. 2013;591:92–94. 10.1113/jphysiol.2012.249060 PMC3832120 23613528

[42] Delestrée N, Manuel M, Iglesias C, Elbasiouny SM, Heckman CJ, Zytnicki D. Adult spinal motoneurones are not hyperexcitable in a mouse model of inherited amyotrophic lateral sclerosis. J Physiol. 2014;592:1687–1703. 24445319 10.1113/jphysiol.2013.265843 PMC3979619

[43] Sareen D, O’Rourke JG, Meera P, et al Targeting RNA foci in iPSC-derived motor neurons from ALS patients with a C9ORF72 repeat expansion. Sci Transl Med. 2013;5:208ra149. 10.1126/scitranslmed.3007529 PMC4090945 24154603

[44] Leroy F, Zytnicki D. Is hyperexcitability really guilty in amyotrophic lateral sclerosis? Neural Regen Res. 2015;10:1413–1415. 26604899 10.4103/1673-5374.165308 PMC4625504

[45] Saxena S, Roselli F, Singh K, et al Neuroprotection through excitability and mTOR required in ALS motoneurons to delay disease and extend survival. Neuron. 2013;80:80–96. 24094105 10.1016/j.neuron.2013.07.027

[46] Mogyoros I, Kiernan MC, Burke D, Bostock H. Strength–duration properties of sensory and motor axons in amyotrophic lateral sclerosis. Brain. 1998;121(Pt 5):851–859. 9619189 10.1093/brain/121.5.851

[47] Lugg A, Schindle M, Sivak A, Tankisi H, Jones KE. Nerve excitability measured with the TROND protocol in amyotrophic lateral sclerosis: A systematic review and meta-analysis. J Neurophysiol. 2023;130:1480–1491. 37910562 10.1152/jn.00174.2023

[48] de Carvalho M, Kiernan MC, Swash M. Fasciculation in amyotrophic lateral sclerosis: Origin and pathophysiological relevance. J Neurol Neurosurg Psychiatry. 2017;88:773–779. 28490504 10.1136/jnnp-2017-315574

[49] Vucic S, Westeneng HJ, Al-Chalabi A, Van Den Berg LH, Talman P, Kiernan MC. Amyotrophic lateral sclerosis as a multi-step process: An Australia population study. Amyotroph Lateral Scler Frontotemporal Degener. 2019;20(7–8):532–537. 31284763 10.1080/21678421.2018.1556697

[50] Tankisi H, Bostock H, Grafe P. A test to determine the site of abnormal neuromuscular refractoriness. Clin Neurophysiol Pract. 2022;7:1–6. 34984248 10.1016/j.cnp.2021.11.001 PMC8693356

[51] Shibuta Y, Shimatani Y, Nodera H, Izumi Y. Increased variability of axonal excitability in amyotrophic lateral sclerosis. Clin Neurophysiol. 2013;124:2046–2053. 23726502 10.1016/j.clinph.2013.02.117

