# Supplementary material for: Revisiting distinct nerve excitability patterns in patients with amyotrophic lateral sclerosis
Source: Brain. 2024 Apr 25;147(8):2842–53. doi: 10.1093/brain/awae131 (PMC11491535; doi:10.1093/brain/awae131)
Supplement: awae131_Supplementary_Data [file awae131_supplementary_data.pdf]

Supplementary material

Retention of principal components

We used two different measures to determine the number of principal components (PC) to retain for analyses. These analyses were performed in each of the 20 imputed datasets (see Statistical analysis section) to minimize the potential effects of imputation bias. First, we calculated Krzanowski's Q^2-statistic[1], that indicates the variance described by the PCA-model after combining the loadings and scores between multiple cross-folds consisting of 80% of all the available observations. This process was repeated 10 times, varying the omitted rows. Consequently, poor (low) Q^2 indicates that the PCA-model only describes noise rather than the true data structure and *vice versa*. As shown in supplemental Figure 1, our obtained Q^2-statistic reaches its highest value reaches a stable value from PC4 or larger. Second, we opted to omit PCs that contributed less than 5% of the total variance (R^2), which we considered unlikely pathophysiological contributors of the observed excitability patterns in ALS. Based on these combined metrics and cut-offs we opted to retain PCs 1-4 for subsequent analyses.

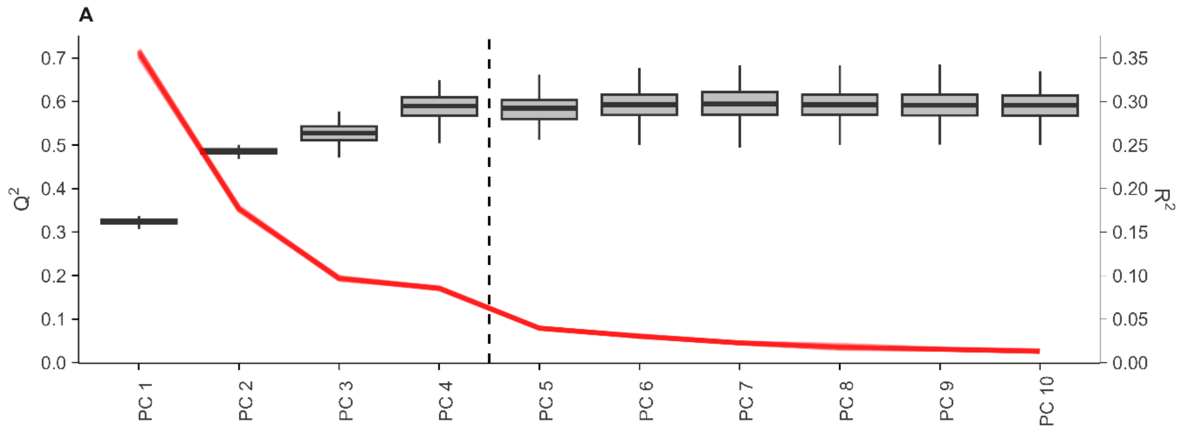

Supplemental Figure 1. Retention of PCs that best describe the underlying data structure. Boxplots and the values on the left y-axis represent Krzanowski's Q^2-statistic that reach an optimum from ≥4 PCs upwards. The red lines and the values on the right y-axis represent the variance explained by each PC; multiple lines are overlayed indicating the similarity of the results from each imputation set.

PC = principal component

Stability of identified excitability patterns

Internal validation was performed to determine the stability of the identified excitability patterns. For this purpose, we performed PCA on random subsets of 20% and 80% of the data, calculating Pearson's correlation coefficient between the PC loadings that describe the transformation of the original recordings (e.g. the "patterns"). This process was repeated 10 times in different subsets. We found that correlations between the loadings were excellent, indicating high robustness of the identified patterns (*R*, median: PC1 = 0.94; PC2 = 0.82; PC3 = 0.67; PC4 = 0.69). Upon visual examination, the excitability patterns obtained from the random subsets of 20% of the data were sufficiently robust to include in the analyses (supplemental Figure 2). The simulation of excitability patterns from prespecified PC-magnitudes is possible via matrix multiplication: $\mathbf{z} = \mathbf{Lx}$, where $\mathbf{L}$ is a matrix with one column per retained PC, $\mathbf{x}$ is a vector with chosen PC-magnitudes and $\mathbf{z}$ is a vector containing the estimated standardized excitability measures.

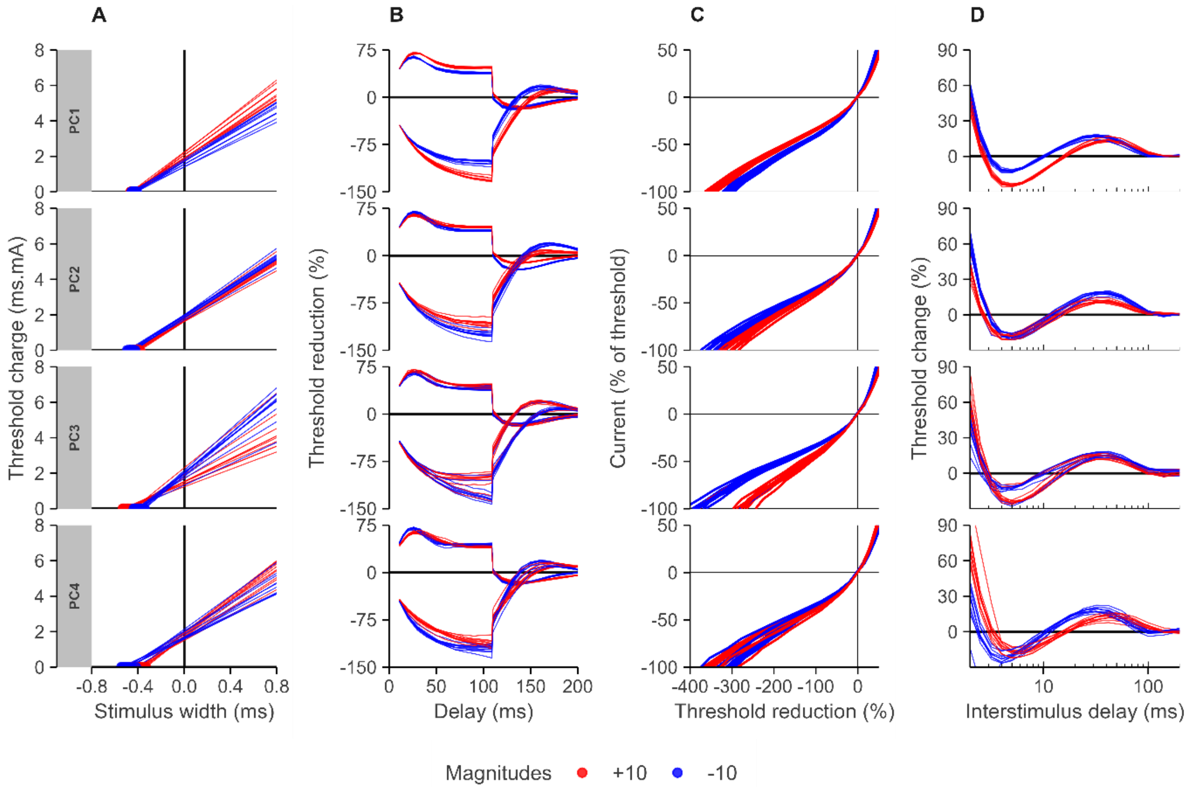

Supplemental Figure 2. Stability between excitability patterns derived from random 20% subsets of the data. Plots represent: **A)** Strength-duration test; **B)** threshold electrotonus; **C)** current-voltage test and; **D)** recovery cycle test. Red lines and blue lines indicate the effects of increasing or decreasing the magnitude of each individual PC by 10.

PC = principal component

Supplemental Table 1: Description of the nerve model parameters and baseline values for optimization obtained from 50 healthy controls

Model parameters	Parameter description	Baseline value
**Channel kinetics**		
Aah	Inactivation rate of sodium channels (ms^{-1})	[0.0215, 0.0108][a]
Aam	Activation rate of sodium channels (ms^{-1})	[1.62, 0.81][a]
Aan	Activation rate of fast potassium channels (ms^{-1})	3.35
Aas	Activation rate of slow potassium channels (ms^{-1})	1.24
Aq	Activation rate of Ih channels (ms^{-1})	0.78
Bah	Voltage of half-inactivation of sodium channels (mV)	[-115.1, -133.1][a]
Bam	Voltage of half-activation of sodium channels (mV)	[-18.5, -36.5][a]
Ban	Voltage of half-activation of fast potassium channels (mV)	-90.8
Bas	Voltage of half-activation of slow potassium channels (mV)	-23.5
Bq	Voltage of half-activation of Ih channels (mV)	-107.3
Cah	Voltage inactivation slope factor of sodium channels (mV)	[15.6, 15.6][a]
Cam	Voltage activation slope factor of sodium channels (mV)	[10.3, 10.3][a]
Can	Voltage activation slope factor of fast potassium channels (mV)	7.7
Cas	Voltage activation slope factor of slow potassium channels (mV)	12.7
Cq	Voltage activation slope factor of Ih channels (mV)	-12.2
**Channel permeability/conductances**		
PNaN	Permeability of sodium channels at the node ($cm^3s^{-1} \times 10^{-9}$)	4.65
PNap	Proportion of persistent sodium channels (%)	1.22
GBB	Barrett-Barrett (nS)	44.4
GH	Ih channel conductance (nS)	6.65
GKfI	Fast internodal potassium conductance (nS)	39.1
GKfN	Fast nodal potassium conductance (nS)	29.8
GKsI	Slow internodal potassium conductance (nS)	0.61
GKsN	Slow nodal potassium conductance (nS)	61.8
GLk	Internodal leak conductance (nS)	3.35
GLkN	Nodal leak conductance (nS)	1.90
**Ion-concentrations and currents**		
IPumpNI	(Inter-)nodal sodium-potassium pump currents (pA)	-0.0151
KI	Intracellular potassium concentration (mM)	155
KO	Extracellular potassium concentration (mM)	4.5
NaI	Intracellular sodium concentration (mM)	9
NaO	Extracellular sodium concentration (mM)	144.2

Temperature was set at 308K. Corresponding resting membrane potentials are: nodal = -81.37mV; internodal = -81.09mV.

[a][transient, persistent]-sodium channel parameter values

References

1. Krzanowski W. Cross-validation in principal component analysis. *Biometrics*. Published online 1987:575-584.
